# Supplementary material for: Isolation and Characterization of a Novel Phage Collection against Avian-Pathogenic Escherichia coli
Source: Microbiol Spectr. 2023 May 4;11(3):e04296-22. doi: 10.1128/spectrum.04296-22 (PMC10269720; doi:10.1128/spectrum.04296-22)
Supplement: Supplemental file 1 — Supplemental material. Download spectrum.04296-22-s0001.pdf, PDF file, 0.5 MB [file spectrum.04296-22-s0001.pdf]

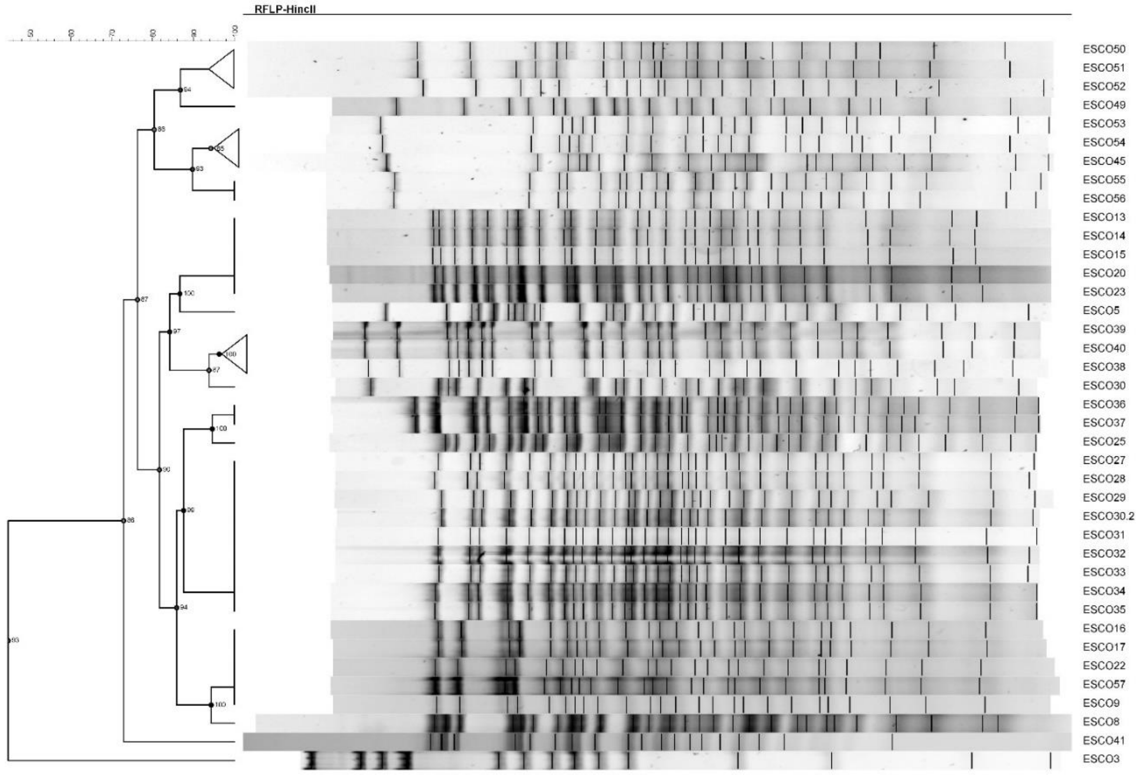

Figure S1: Dendrogram of restriction fragment length polymorphism (RFLP) profile of DNA of isolated phages digested with HincII (generated by UPGMA and based on Dice coefficient).

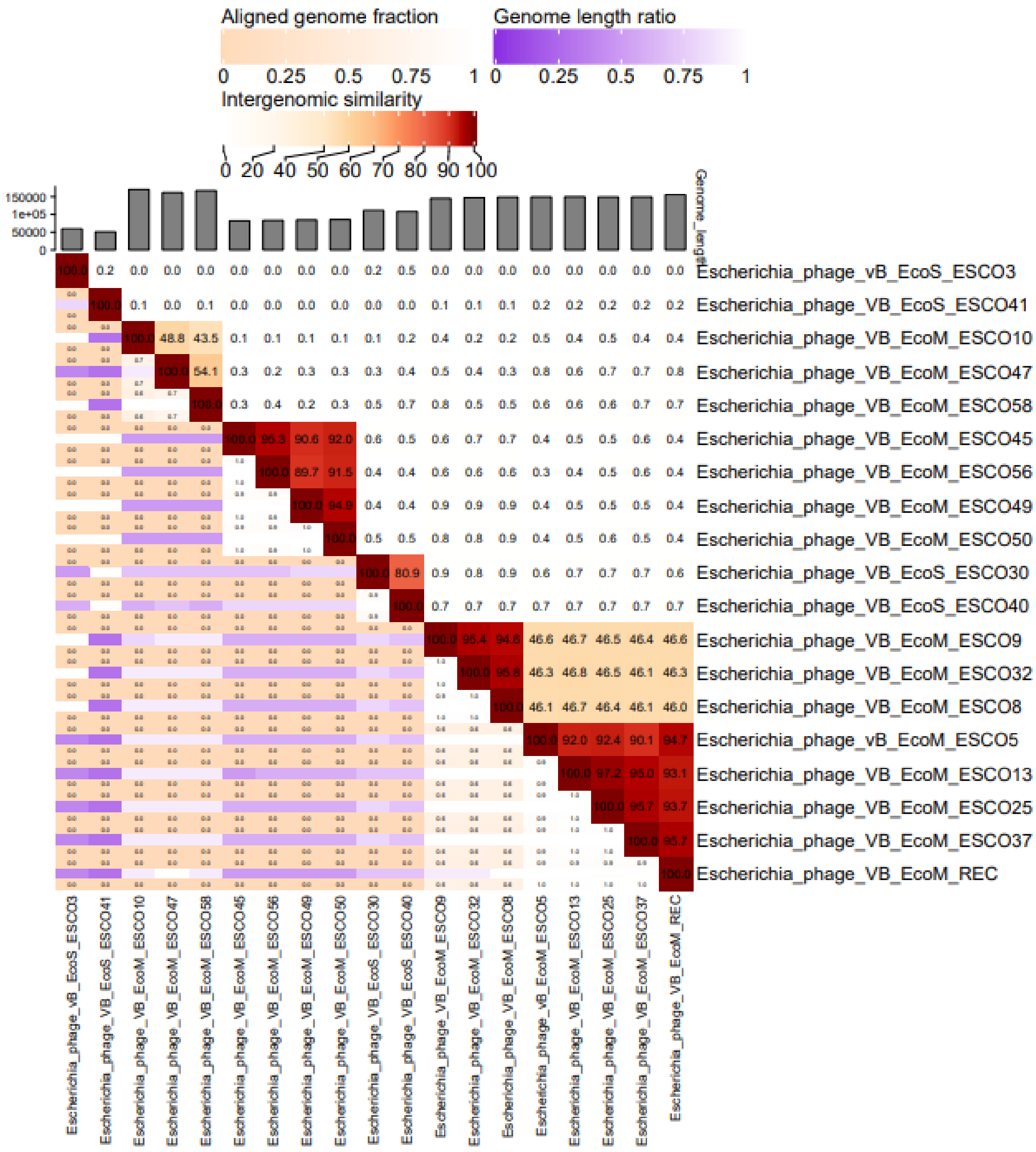

Figure S2: Heat map generated by VIRIDIC. The left side shows the intergenomic similarity between the 19 genomes using a gradient in red, with darker colors showing a higher percent of intergenomic similarity. The right half represents three different values, the aligned genome fraction for the genome found in the row (on the bottom), genome length ratio (purple gradient), and the aligned genome fraction (on the top) for the genome found on the column, all ranked from 0 to 1, with 1 represented in white.

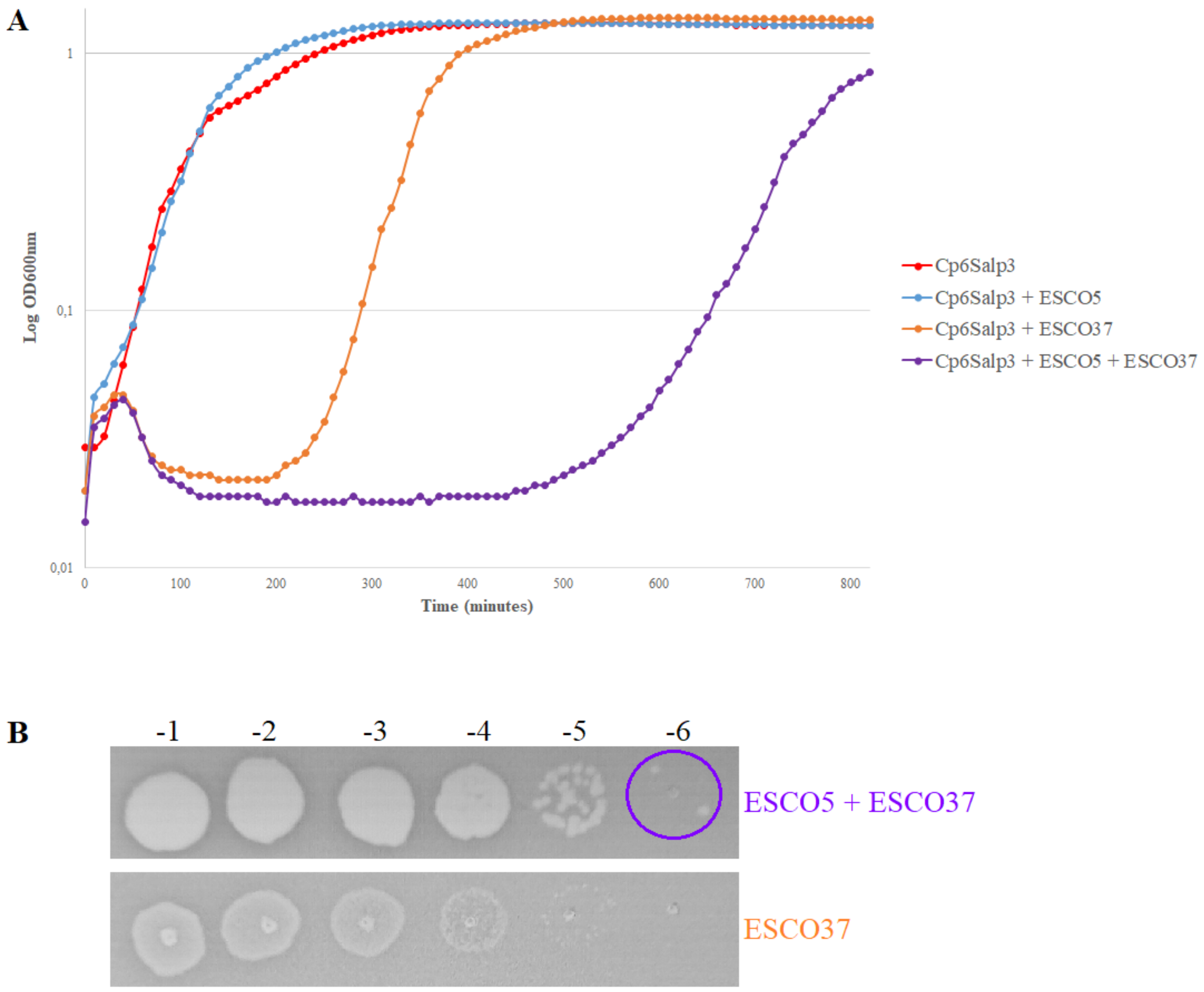

Figure S3: *In vitro* lytic activity of a two-phage cocktail against APEC BEN5217 strain showed the appearance of a new phage. (A) An overnight bacterial culture was diluted and infected with ESCO5 (blue curve) or ESCO37 (purple curve) or both phages (green curve) at MOI 0.1. Non-phage-treated bacterial cultures were used as a positive control (red curve). Bacterial growth at 37°C was recorded by monitoring OD600nm every 10 minutes during 24 h. (B) Supernatants of 24 h cultures of Cp6Salp3 with ESCO37 alone and Cp6Salp3 with phages ESCO5 and ESCO37 were spotted on a Cp6Salp3 bacterial lawn to observe the lysis plaques. Clearer and bigger plaques (purple circled) were observed when Cp6Salp3 was infected with ESCO5 and ESCO37 in combination compared to infection with ESCO37 alone.

Table S1: Head and tail dimension of isolated coliphages.

		Mean ± SD		
**Phage name**	**Morphology type**	**Head size (nm)**	**Tail size (nm)**	**Number of measurements**
vB_EcoM_ESCO8	Myovirus	81.8 ± 5.0	104.1 ± 2.9	7
vB_EcoM_ESCO9	Myovirus	68.5 ± 9.1	99.2 ± 9.1	16
vB_EcoM_ESCO32	Myovirus	93.6 ± 5.9	106.0 ± 9.9	8
vB_EcoM_ESCO5	Myovirus	87.2 ± 4.2	93.9 ± 11.4	23
vB_EcoM_ESCO13	Myovirus	60.1 ± 4.7	109.1 ± 8.3	9
vB_EcoM_ESCO25	Myovirus	76.2 ± 7.7	105.7 ± 12.0	4
vB_EcoM_ESCO37	Myovirus	76.0 ± 4.6	123.0 ± 14.0	6
vB_EcoM_REC	Myovirus	78.5 ± 5.1	75.0 ± 23.2	14
vB_EcoM_ESCO45	Myovirus	70.6 ± 2.8	118.8 ± 4.1	10
vB_EcoM_ESCO49	Myovirus	68.3 ± 4.0	117.6 ± 6.7	11
vB_EcoM_ESCO50	Myovirus	71.9 ± 4.4	117.7 ± 5.9	10
vB_EcoM_ESCO56	Myovirus	71.0 ± 3.9	120.8 ± 4.3	6
vB_EcoM_ESCO10	Myovirus	112.5 ± 10.9	115.4 ± 6.4	8
vB_EcoM_ESCO47	Myovirus	91.7 ± 7.0	116.0 ± 7.2	5
vB_EcoM_ESCO58	Myovirus	101.9 ± 6.8	119.8 ± 10.3	23
vB_EcoS_ESCO3	Siphovirus	77.1 ± 5.6	181.7 ± 10.0	23
vB_EcoS_ESCO30	Siphovirus	71.9 ± 2.1	181.6 ± 26.5	6
vB_EcoS_ESCO40	Siphovirus	67.5 ± 3.3	183.8 ± 20.5	5
vB_EcoS_ESCO41	Siphovirus	62.9 ± 4.7	156.2 ± 13.3	6

Table S2: Details of strains used in this study.

Species	Laboratory name	Serogroup	Origin	Reference
Escherichia coli	BEN36 [b]	O5	APEC	This study
	BEN83 [b]	O8	APEC	This study
	BEN88 [b]	O5	APEC	This study
	BEN248 [b]	O5	APEC	This study
	BEN259 [b]	O5	APEC	This study
	BEN349 [a]	O8	APEC	This study
	BEN536 [b]	O78	APEC	This study
	BEN984 [b]	O5	APEC	This study
	BEN1063 [b]	O8	APEC	This study
	BEN1274 [b]	O8	APEC	This study
	BEN1403 [b]	O8	APEC	This study
	BEN1410 [b]	O8	APEC	This study
	BEN1487 [a, b]	O18	APEC	This study
	BEN1589 [b]	O1	APEC	This study
	BEN2908 [a, b]	O2	APEC	PRJEB35043
	BEN3328 [a, b]	O78	APEC	This study
	BEN3388 [b]	O2	APEC	This study
	BEN3546 [a]	O78	APEC	This study
	BEN3573 [a, b]	O78	APEC	This study
	BEN3685 [b]	O2	APEC	This study
	BEN3693 [a]	O88	APEC	This study
	BEN3801 [b]	O18	APEC	This study
	BEN3813 [b]	O78	APEC	This study
	BEN3843 [b]	O1	APEC	This study
	BEN3992 [a, b]	O18	APEC	This study
	BEN4121 [b]	O18	APEC	This study
	BEN4219 [a]	O78	APEC	This study
	BEN4234 [b]	O1	APEC	This study
	BEN4237 [b]	O18	APEC	This study
	BEN4311 [a, b]	O1	APEC	This study
	BEN4358 [a, b]	O2	APEC	This study
	BEN4467 [a]	O2	APEC	This study
	BEN4481 [b]	O78	APEC	This study
	BEN4484 [a]	O88	APEC	This study
	BEN5040 [a]	O25	APEC	This study
	BEN5202 [a, b]	O2	APEC	LR740758.1
	Cp6Salp3 [a, b]	O1	APEC	JYED01000000
	DH5α [a, b]	O-	Laboratory strain	CP026085.1
	MG1655 [b]	O-	Commensal	U00096.2
Salmonella enterica subsp. *enterica*	LA5 [b]	Enteritidis		CAGR00000000.2
	1281 [b]	Infantis		This study
	ATCC14028 [b]	Typhimurium		CP001363

[a] Strains used in phage isolation screening.

[b] Strains used in host range determination.
